# Stability of Cross-Feeding Polymorphisms in Microbial Communities

**DOI:** 10.1371/journal.pcbi.1005269

**Published:** 2016-12-30

**Authors:** Ivana Gudelj, Margie Kinnersley, Peter Rashkov, Karen Schmidt, Frank Rosenzweig

**Affiliations:** 1 Biosciences, University of Exeter, Exeter, United Kingdom; 2 Division of Biological Sciences, University of Montana, Missoula, Montana, United States of America; 3 School of Biological Sciences, Georgia Institute of Technology, Atlanta, Georgia, United States of America; Massey University, NEW ZEALAND

## Abstract

Cross-feeding, a relationship wherein one organism consumes metabolites excreted by another, is a ubiquitous feature of natural and clinically-relevant microbial communities and could be a key factor promoting diversity in extreme and/or nutrient-poor environments. However, it remains unclear how readily cross-feeding interactions form, and therefore our ability to predict their emergence is limited. In this paper we developed a mathematical model parameterized using data from the biochemistry and ecology of an *E*. *coli* cross-feeding laboratory system. The model accurately captures short-term dynamics of the two competitors that have been observed empirically and we use it to systematically explore the stability of cross-feeding interactions for a range of environmental conditions. We find that our simple system can display complex dynamics including multi-stable behavior separated by a critical point. Therefore whether cross-feeding interactions form depends on the complex interplay between density and frequency of the competitors as well as on the concentration of resources in the environment. Moreover, we find that subtly different environmental conditions can lead to dramatically different results regarding the establishment of cross-feeding, which could explain the apparently unpredictable between-population differences in experimental outcomes. We argue that mathematical models are essential tools for disentangling the complexities of cross-feeding interactions.

## Author Summary

Simple environments, even those used in laboratory experimental evolution, have proven vastly richer than originally thought, capable of generating and supporting genetic and phenotypic diversity. This was not foreseen by Gause’s seminal competitive exclusion theory, which predicted that simple single niche environments cannot support diversity. We now know that cross-feeding interactions can be a major driver of diversity maintenance in simple environments. Cross-feeding, a relationship wherein one organism consumes metabolites excreted by another, is a ubiquitous feature of natural and clinically-relevant microbial communities and even tumour cell populations. However, it remains unclear how readily such relationships form, and therefore our ability to predict their emergence is limited. Here we developed a mathematical model of cross-feeding and find that this system can display complex dynamics including multi-stable behaviour separated by a critical point. Therefore, the emergence of cross-feeding depends on complex interplay between density and frequency of competitors. Moreover we predict that small changes in environmental conditions can cause abrupt and irreversible shifts from cross-feeding permissive to cross-feeding prohibitive states. We argue that mathematical models are essential tools for disentangling the complexities of cross-feeding interactions.

## Introduction

Why are microbial communities so diverse, and how is this diversity maintained? These questions have shaped research in microbial ecology for decades and mathematical models have been instrumental in providing answers. In particular, Gause’s theory of competitive exclusion [1], popularized by Hardin [2], has deeply influenced our understanding of the type of environments that can support organismal diversity. This theory states that simple environments containing a single resource niche can only support one competitor; therefore the search for mechanisms supporting diversity was, for years, focused around complex environments. This ecological principle was supported by an evolutionary principle articulated by Muller [3], who postulated that a large asexual population evolving in a simple environment should evolve by periodic selection of successively fitter clones, each going to fixation and resulting in clonal replacement.

However in the late 1980s, a seminal experimental work put the spotlight back onto simple constant environments by demonstrating, quite unexpectedly, that such environments could both generate and support genetic diversity [4]. A population of *E*. *coli* initiated from a single clone and cultured under constant glucose limitation for over 750 generations became stably polymorphic, with clones differing significantly in their glucose uptake kinetics as well as in their maximum specific growth rates and yield under non-limiting conditions. A subsequent study [5] demonstrated that the mechanism by which polymorphism was stably maintained in this population was cross-feeding, an independent relationship wherein one genotype consumes metabolites excreted by another. Specifically, Rosenzweig *et al*. found that the clone with the highest uptake kinetics of the primary limiting resource excreted metabolites that created alternative secondary resource niches on which other clones could specialize. While this finding did not contradict Gause and Muller, it showed that microorganisms could readily move outside the assumptions of the competitive exclusion principle. Interestingly, even though each clone specialized on a different niche, both retained the capacity to assimilate either resource, albeit at very different rates.

The construction of multiple niches where initially there was only one, arose as the consequence of a metabolic trade-off whereby organisms can convert available limiting resources into energy either slowly but efficiently or rapidly but wastefully [5]. When the primary resource is utilized wastefully, the resulting waste product can serve as a secondary energy source. This rate-efficiency trade-off is considered to be a thermodynamic [6] and biophysical [7] necessity and has been observed in a wide range of microorganisms (as discussed in [8]).

It is becoming clear that cross-feeding, whereby one strain or species consumes metabolites produced by another, is a pervasive feature of microbial communities in nature [9, 10]. Indeed, the advent of cross-feeding opens the door to other more complex interactions such as syntrophy, wherein a consumer strain releases metabolites that are useful to the producer. Syntrophic interactions, which demonstrably benefit both partners, are ubiquitous among free-living bacteria in pristine [11] and human-impacted environments [12, 13], and have now been studied systematically in synthetic communities [14]. These types of interactions may also be at work in clinically relevant settings where cells reproduce asexually. For example, chronic bacterial infections originating from a single clone become genetically heterogeneous [15, 16], and in some cases this heterogeneity appears to be supported by syntrophic interactions [17]. Extreme genetic heterogeneity is also a characteristic feature of evolving tumors [18]. As tumors are known to carry out aerobic glycolysis [19] leading to a release of overflow metabolites [20], this may create opportunities for subpopulations to follow independent evolutionary trajectories reinforced by cross-feeding [21].

What is not clear is how readily cross-feeding interactions form. Even in environments known to favor cross-feeding, the emergence of such interactions is not consistently observed. For example, a study similar to [5] found that six out of twelve *E*. *coli* populations did not develop cross-feeding polymorphisms while the other six did [22]. An unrelated study [23] followed an initially clonal population of *E*. *coli* in glucose limited continuous culture over 100 generations. This population radiated into multiple phenotypic clusters each of which exhibited variations in global regulation, metabolic strategies, surface properties and nutrient permeability pathways. However, in this instance diversity resulted from a mixture of mechanisms including mutation-selection balance, frequency dependent selection, trade-offs and regulatory degeneracies [23, 24].

A possible clue to the apparent instability of cross-feeding interactions may lie in the observed density-dependent dynamics between primary resource specialist and secondary resource specialist clones [5]. In particular, the equilibrium frequencies of coexisting clones were strongly dependent on the total population densities, with high population densities favouring primary resource specialists.

To investigate this phenomenon we here develop a mathematical model that describes a cross-feeding interaction between two microbial strains growing and competing in a spatially homogeneous environment that contains a single limiting resource. This model is parameterized using data from the biochemistry and ecology of two *E*. *coli* isolates known to support cross-feeding interactions [5]. We show that the model can qualitatively capture the empirically observed short-term dynamics of the two competitors. We then consider the long-term population dynamics and ask under what environmental conditions is this cross-feeding maintained. We find that this seemingly simple system can display complex dynamic behaviors, which depend on the density and frequency of the competitors as well as on the concentration of available resources.

At sufficiently low resource concentrations the strain that consumes the primary resource, outcompetes the strain that consumes the secondary resource, which is a byproduct of primary resource metabolism. At intermediate concentrations of the primary resource, either strain can outcompete the other depending on densities of the competitors and their initial frequencies. This can be explained by the fact that while each strain specializes on a different resource, they retain the capacity to utilize both. Finally, at sufficiently high concentrations of the primary resource three outcomes are possible: either strain outcompetes the other or they coexist through a cross-feeding interaction. Again, this tri-stable outcome is density and frequency dependent. Therefore whether cross-feeding emerges in such a system depends not only on the primary resource being sufficiently abundant but also on the size of the initial population and the frequency of competitors within that population.

These findings illuminate the dynamic nature of cross-feeding interactions and demonstrate the utility of mathematical models in predicting conditions under which cross-feeding can become established and persist. Because cross-feeding leads to increased biocomplexity, knowledge of how cross-feeding arises in the lab deepens our understanding of the mechanisms by which asexual populations diversify in nature, whether in the context of soils and sediments, chronic infections or evolving tumors.

## Materials and Methods

### The mathematical model

**Fig 1:**
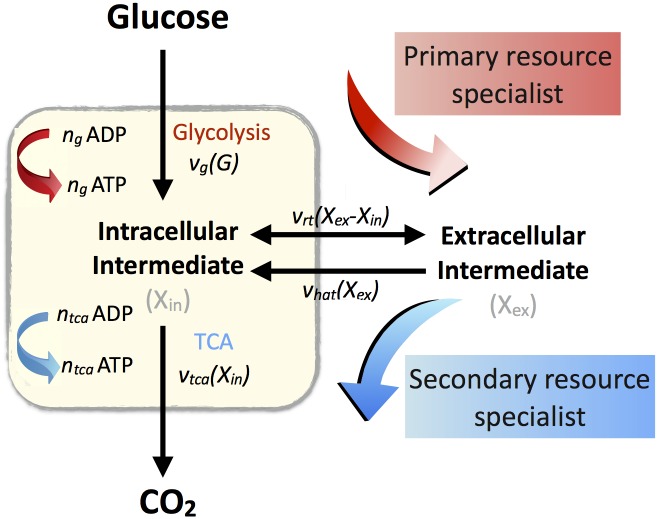
Schematic model of cross-feeding interactions. One strain specializes on the primary limiting resource (glucose) rapidly but inefficiently, excreting a metabolic intermediate (acetate) as a waste product. This waste product serves as a secondary resource for another strain to specialize on. Note that the two strains can utilize both the primary and the secondary resource.

To investigate the stability of a cross-feeding interaction, we developed a chemostat model of competition for a simple sugar. For simplicity, we model the catabolism of sugar and its intermediates as a two-reaction process [25] corresponding to glycolysis and the tricarboxylic acid (TCA) cycle (see Fig 1 for the pathway schematic and S1 Supporting Information for model details). In the first reaction, sugar is taken from the environment and partially oxidized to form an intracellular metabolic intermediate. In the second reaction, the intracellular metabolic intermediate is either completely oxidized to form CO_2_, or excreted out of the cell as an extracellular metabolic intermediate. The extracellular metabolic intermediate can be subsequently taken up into the cell and the excretion/uptake of the metabolic intermediate shows saturating enzyme kinetics [5]. We also assume that both glycolysis and TCA reactions show saturating enzyme kinetics, that the rate of cell growth is proportional to the rate of ATP production [26] according to a proportionality constant *G* and that intracellular metabolic intermediate imposes an inhibitory cost to growth [27] denoted by a function *c*.

Let *S* denote the concentration of a single limiting sugar in the environment while *X*_*in*_ and *X*_*ex*_ denote the concentration of the intracellular and extracellular metabolic intermediates, respectively. The concentration of *S*, *X*_*in*_ and *X*_*ex*_ is measured in μmol/L. In addition we consider two competitor strains whose densities are denoted by *N*_*1*_ and *N*_*2*_ measured as cells/L. Both strains can utilize *S* and *X*_*ex*_ for growth but with the following important difference: *N*_*1*_ specialises on *S*, while *N*_*2*_ specialises on *X*_*ex*_. We use *v*_*g*,*i*_ and *v*_*tca*,*i*_ to denote the rate of glucose uptake and TCA cycle respectively while *v*_*rt*,*i*_ denotes the rate of reversible transport of the metabolic intermediate in and out of the cell, where the subscript *i = 1*,*2* refers to the two strains. The constants *n*_*g*_ and *n*_*TCA*_ denote the yield of ATP from glycolysis and the TCA cycle, respectively.

In addition, by the very nature of cross-feeding, the strain specializing on the extracellular metabolic intermediate (*X*_*ex*_) possesses a high affinity enzyme for the uptake of *X*_*ex*_ and the rate of this enzyme’s kinetics, which does not use ATP as cofactor, is denoted by *v*_*hat*_. Note that this term is not present in [25] as their model does not explicitly consider cross-feeding interactions.

The competition dynamics of the two strains in the chemostat are described as

$$\begin{array}{ll} \frac{dS}{dt} & =D\left(S_{0}-S\right)-v_{g,1}\left(S\right)N_{1}-v_{g,2}\left(S\right)N_{2}\\ \frac{dX_{ex}}{dt} & =v_{rt,1}\left(X_{in,1}-X_{ex}\right)N_{1}+(v_{rt,2}\left(X_{in,2}-X_{ex}\right)-v_{hat}\left(X_{ex}\right))N_{2}-DX_{ex}\\ \frac{dN_{1}}{dt} & =G\left(v_{g,1}\left(S\right)n_{g}+v_{tca,1}\left(X_{in,1}\right)n_{tca}\right)c_{1}\left(X_{in,1}\right)N_{1}-DN_{1}\\ \frac{dN_{2}}{dt} & =G\left(v_{g,2}\left(S\right)n_{g}+v_{tca,2}\left(X_{in,2}\right)n_{tca}\right)c_{2}\left(X_{in,2}\right)N_{2}-DN_{2}\\ \frac{dX_{in,1}}{dt} & =\left(2v_{g,1}\left(S\right)-v_{tca,1}\left(X_{in,1}\right)-v_{rt,1}\left(X_{in,1}-X_{ex}\right)\right)N_{1}-DX_{in,1}\\ \frac{dX_{in,2}}{dt} & =\left(2v_{g,2}\left(S\right)-v_{tca,2}\left(X_{in,2}\right)-v_{rt,2}\left(X_{in,2}-X_{ex}\right)+v_{hat}\left(X_{ex}\right)\right)N_{2}-DX_{in,2} \end{array}$$ (1)

where *D* represents the dilution rate and *S*_*0*_ is the concentration of resources in the input vessel. Note that in the last two equations a factor of 2 in front of the glucose uptake rate *v*_*g*,*i*_ denotes a stoichiometric coefficient signifying that one hexose molecule is converted into two triose molecules.

We parameterized this model using data on the biochemistry and ecology of *E*. *coli* using two cross-feeding strains: CV103 and CV101 grown in glucose-limited chemostats [5]. Strain CV103 best scavenges but incompletely metabolizes the primary (limiting) resource, glucose, while CV101 consumes a secondary resource, acetate, which is the overflow metabolite produced by CV103 (see S1 Supporting Information for details of parameter values and the corresponding units of measurement).

## Results

### The simple model accurately captures empirical observations

Our parameterized system shows that CV103 is primarily a “fermenter” while CV101 is a “respirer” (S1 Supporting Information). This is consistent with the findings of [28] and is further confirmed by measuring cell redox balance (NADH/NAD+) in chemostat monocultures of the two cell types (S1 Supporting Information).

**Fig 2:**
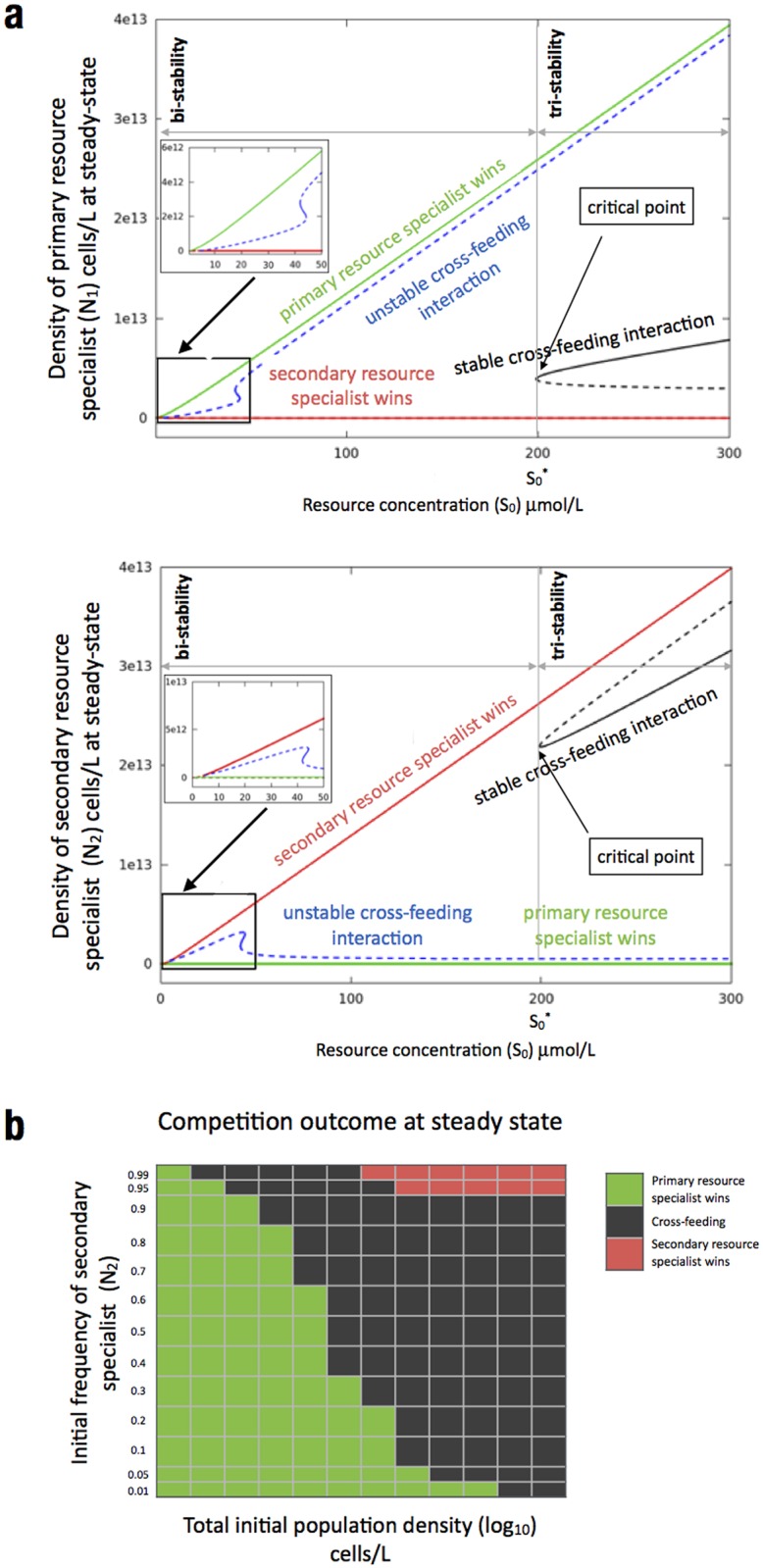
Generalizing competition outcomes predicted by the model for a range of environmental conditions. **(a)** A bifurcation diagram showing model predictions regarding all possible long-term competition outcomes as a function of glucose concentration in the input vessel (S_0_), regardless of the initial population densities and frequencies. Densities [cells/L] of the primary and secondary resource specialist at steady state are shown in the top and bottom panel, respectively. Full lines denote locally stable steady states while dashed lines denote unstable steady states; black boxes near the origins guide the eye to the regions where for sufficiently low S_0_ the following can happen: no competitor can survive, only glucose specialist can survive or cross-feeding is possible. **(b)** The long-term competition outcome as a function of different initial population densities and frequencies for a fixed glucose concentration in the input vessel (S_0_ = 350 μm/L).

Experimental data on the growth of CV101 and CV103 in mixed cultures of the chemostat [5] showed that the two strains can be maintained over 30 generations. Our model successfully captures this outcome as it contains a stable cross-feeding steady state in which the two strains coexist in the long-term (Fig 2).

**Fig 3:**
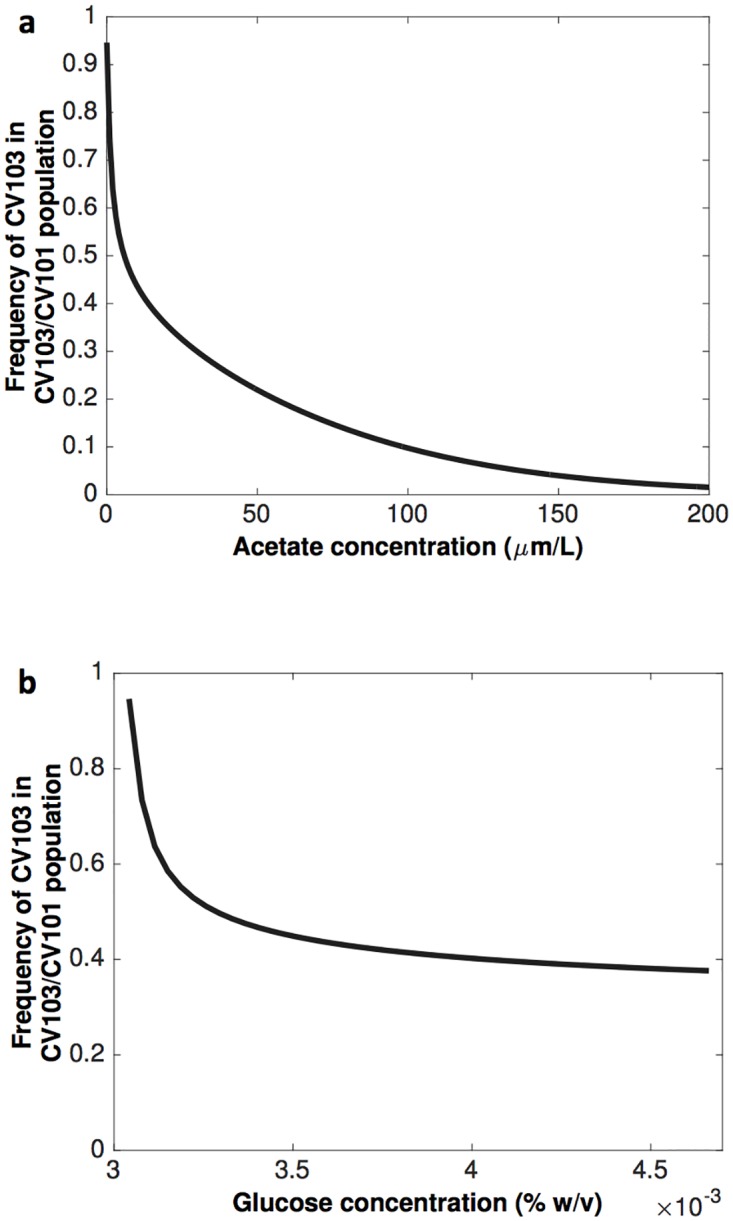
The mathematical model qualitatively captures previously observed experimental findings [5]. **(a)** The frequencies of CV103 and CV101 are affected by the level of acetate in the medium with the addition of acetate [μmol/L] in the culture leading to a decrease in the frequency of CV103; **(b)** The frequencies of CV103 and CV101 are affected by the total population density with higher densities achieved by increasing the resource concentration in the input vessel, S_0_ [% weight/volume]. This increase in S_0_ provides a growth advantage for CV101 thereby decreasing the frequency of CV103. In both **(a)** and **(b)** simulations were conducted over 30 generations.

Since the primary resource consumer (CV103) specializes on glucose [4] while the secondary resource consumer (CV101) specializes on acetate secreted by CV103 [5], the outcome of their interaction depends on the levels of acetate in the culture. In particular, when the two strains were grown together in continuous mixed cultures the frequency of the competitors after 30 generations was strongly affected by the addition of acetate to the medium [5]. The addition of acetate into the culture leads to a decrease in the frequency of the primary resource specialist, CV103 and the model is able to qualitatively capture this result, as illustrated in Fig 3a.

Finally, previous work showed that the frequency of competitors over 30 generations strongly depended on population density [5] with higher population densities achieved by increasing the concentration of the incoming limiting carbon source into the environment, providing growth advantage to the secondary resource specialist (CV101). Again, our model is capable of qualitatively capturing this outcome as shown in Fig 3b.

The simplicity of the model assumptions enables a systematic exploration of the environmental conditions and their effects on the stability of cross-feeding interactions as described next.

### Exploring stability of cross-feeding interactions

Having established that our metabolic population model is able to qualitatively capture the observed data, we ask a broader question: How stable are cross-feeding interactions for a wider variety of environmental conditions?

We address this question by carrying out a bifurcation analysis, which is classically used in mathematics to investigate the behavior of systems of differential equations like the one described in model (1). Moreover, bifurcation theory is frequently used to study competitive interactions in biology [29–31]. The results of the analysis are presented as a bifurcation diagram (Fig 2a), which shows steady states of our model as a function of a bifurcation parameter, namely the incoming primary resource concentration. Stable steady states are represented with a solid line and unstable states with a dashed line. The benefit of performing a bifurcation analysis is that we are able to systematically explore a large set of parameter values determining the steady-state outcomes of our model for all possible initial population frequencies and densities.

Our analysis uncovers a complex picture (Fig 2a) arising from relatively simple assumptions regarding metabolic interactions of the competitors (Fig 1). In particular, for sufficiently high resource concentrations our system exhibits tri-stability which means that for a given resource concentration there are three possible competition outcomes depending on the initial population density and frequency of the two competitors. In that case, either of the two competitors can outcompete the other or both can coexist in a cross-feeding interaction (Fig 2a, tri-stability region).

**Fig 4:**
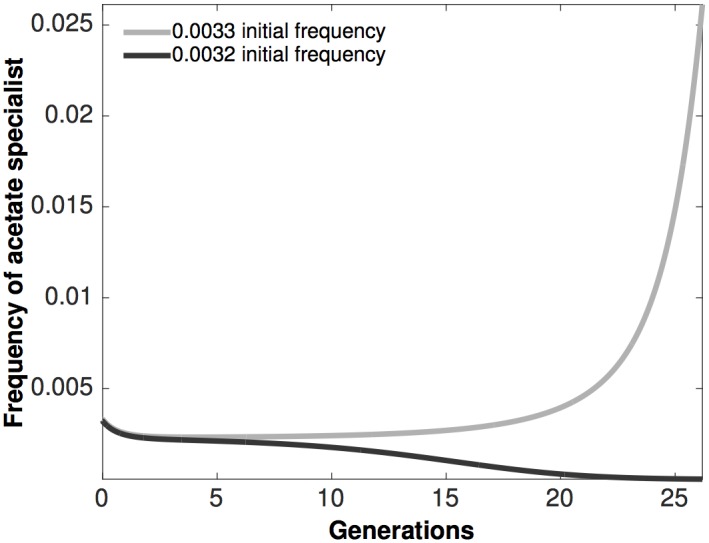
An example of bi-stable dynamics within the model system. The total initial population density is at the maximal level supported in 0.025% glucose environment. When the initial frequency of acetate specialists is 0.0033, the strain is able to increase in frequency and establish cross-feeding interactions with the glucose specialist. When the initial frequency of acetate specialist is 0.0032, the strain is not maintained within the population.

By definition, a bifurcation diagram identifies all possible steady-states and their stability as a function of the bifurcation parameter, regardless of the initial population densities and frequencies. An illustration of the long-term model outcomes as a function of different initial population frequencies and densities for a fixed concentration of the incoming primary resource is shown in Fig 2b. To further illustrate the multi-stable dynamics of our system we plot time course solutions of our system (1) for different initial conditions. In particular we capture the hallmark of multi-stability whereby a small change in the initial conditions can cause a qualitative change in the behavior of the system. For example, the fitness of an initially rare secondary resource specialist (*N*_*2*_) is frequency dependent (Fig 4), with small differences in initial frequencies giving rise to two outcomes: *N*_*2*_ decreases in frequency leading to its exclusion from the environment or *N*_*2*_ increases in frequency leading to coexistence between the competitors.

For intermediate primary resource concentrations the system exhibits bi-stability where two outcomes are possible: either the primary resource specialist outcompetes the secondary resource specialist or vice versa. Which one is the case depends on the initial densities and frequencies (Fig 2a, bi-stability region).

For a certain range of low primary resource concentrations, the system again exhibits tri-stability where in addition to either strain outcompeting the other, cross-feeding is also possible (Fig 2a, boxed plots). However, due to the narrow range of resource concentrations supporting cross-feeding in this case, it is unlikely that such outcome would be observed empirically.

Decreasing further the primary resource concentration we found that only the primary resource specialist can persist (Fig 2a, boxed plots). In addition, if the resource concentration in the input vessel is below a certain threshold, no competitor can survive; however this occurs at primary resource concentrations that are not clearly visible on the bifurcation diagram.

Finally we note that the qualitative nature of competition outcomes presented in the bifurcation diagram in Fig 2a, including the existence of multi-stable steady states, is robust to changes in the model parameter values as discussed in S1 Supporting Information.

Next we ask what are the key biological mechanisms that enable cross-feeding in our model? It has been shown [32] that in simple environments cross-feeding is possible if the following two conditions are satisfied. First, there should exist a trade-off between uptake efficiencies on the primary and secondary resource niches and second that this trade-off takes a convex form. The trade-off convexity implies decreasingly costly investment [33] whereby initial improvement in uptake efficiency on a given resource leads to substantial decrease in uptake efficiency on the alternative resource, with subsequent improvements taking place at little or no additional costs.

In general, it is known that the form of a trade-off determines the outcome of competition [34–36]. So is the existence of a convex trade-off between utilization of different resources enabling cross-feeding in our system? While there is some experimental evidence for the existence of such a trade-off [5, 37, 38] its form has yet to be determined empirically. In fact, we argue that in our system this trade-off does not conform to a convex geometry for the following reasons. The secondary resource specialist abundantly expresses a high affinity enzyme (acetyl CoA synthetase) for the uptake of the secondary resource, acetate [5]. Doing so it substantially improves its ability to utilize this resource relative to the primary resource specialist which negligibly expresses this enzyme. However, the production of this high affinity enzyme by the secondary resource specialist comes at a relatively small cost in terms of its ability to utilize the primary resource; this can be observed by comparing the maximum glucose uptake rates of the two strains (S1 Supporting Information) and is a signature of a concave rather than a convex trade-off form [33].

Our model therefore shows that a convex trade-off between utilization of different resources is not a prerequisite for cross-feeding to be observed. Instead we highlight the importance of an additional mechanism for maintaining cross-feeding: a trade-off between rate and yield of primary resource utilization. Considered a thermodynamic [6] and biophysical (Meyer *et al*. 2015) necessity, the rate-yield trade-off has been documented across microbial species [7, 8].

In *E*. *coli* acetate overflow results not only in lost carbon, but also in production of a growth inhibitor [39] In our model the rate-yield trade-off is mediated by the glucose specialist secreting this toxic metabolite, acetate, which forms the secondary resource niche. For example, by being a “fermenter” (S1 Supporting Information), the glucose specialist has a faster uptake rate of glucose than the acetate specialist who is a “respirer” (S1 Supporting Information). On the other hand, the glucose specialist has a reduced yield of ATP production relative to the acetate specialist (S1 Supporting Information). Further, the acetate specialist reduces the concentration of this toxic metabolic intermediate in the environment, thus benefiting the glucose specialist.

However, removing the assumption that a secreted extracellular metabolite imposes a cost to growth does not destroy the existence of cross-feeding steady state. In this case higher concentration of the primary resource is required for cross-feeding to be observed.

In the absence of a trade-off between utilization of different resources, the presence of the rate-yield trade-off does not guarantee coexistence of a respirer and a fermenter in simple environments, as shown in [25]. This allows us to conclude that the presence of *both* trade-offs is crucial for the maintenance of cross-feeding and for the complex density and frequency dynamics to be observed.

Moreover, our model shows that convexity of the trade-off between utilization of different resources is not necessary to maintain cross-feeding, as originally thought [32].

## Discussion

Simple environments, even those used in laboratory experimental evolution, have proven vastly richer than originally thought, capable of generating and supporting genetic and phenotypic diversity. This was not foreseen by the competitive exclusion principle [1], which predicted that simple single niche environments cannot support diversity. A series of seminal laboratory studies [4, 5] identified cross-feeding interactions between strains originating from a common ancestor as a diversity maintenance mechanism. Importantly, these studies showed that due to metabolic complexities within microorganisms, initially simple environments can quickly increase in complexity via the emergence of new resource niches, thus violating the simple assumptions of the competitive exclusion principle.

We now know that such cross-feeding interactions are not just a feature of laboratory systems but are ubiquitous in natural microbial communities, microbial disease infections and even tumour cell populations. However, the precise conditions that promote or limit the emergence of diversity in simple environments are not well understood.

To this end we developed a mathematical model that tracks, in time, the dynamics between two microbial strains: a strain that wastefully consumes the primary limiting resource, excreting a secondary metabolite on which another strain specializes. The complex metabolic processes were simplified and represented by a two-step metabolic model (Fig 1); a similar framework has previously been successful in capturing a range of empirically observed microbial interactions [25].

Our model was parameterized using a well-established *E*. *coli* cross-feeding system (Rosenzweig et al. [5]), and we showed it can qualitatively capture key experimental observations. In particular, cross-feeding interactions can be maintained in the system (Fig 2) and are influenced by the level of secondary metabolite (acetate) excreted into the environment (Fig 3a). As expected, the level of acetate in the environment is positively correlated with the availability of the primary resource (glucose); the higher the concentration of glucose, the higher the concentration of acetate that is excreted by the primary resource specialist (S1 Supporting Information). Therefore high resource concentrations will favour the secondary resource specialist feeding on acetate (Fig 3b).

The relative simplicity of the mathematical model allowed us to systematically explore the stability of cross-feeding interactions across a range of parameters and initial conditions (Fig 2). We uncovered complex dynamics involving bi- and tri-stable steady states as well as the existence of critical points. This indicates that having a sufficiently high concentration of a primary resource, which gives rise to high concentrations of a secondary metabolite in the environment, does not guarantee that cross-feeding will be maintained. Instead, the emergence of cross-feeding in our model was frequency-and density-dependent, which is of particular relevance when one considers the type and order of specific mutants arising in an evolving system as we now discuss.

In the empirical system motivating our theoretical model, cross-feeding emerged when a founder *E*. *coli* population was grown on a single limiting resource, glucose. Genetic changes that influence glucose assimilation arose early in this population [28], and similar changes appear to have arisen in replicate populations [4]. Mutants with improved glucose assimilation, termed glucose specialists, were found to be the “engine” generating diversity, not because they produce new genetic variants, but because they create new niches via the wasteful consumption of the limiting primary resource, glucose [28]. The creation of new niches in the form of overflow metabolites opens the door to specialists that can profitably use these resources.

How often do such interactions arise? A small colony variant (SCV) phenotype typical of the glucose specialist arose in 11 of 15 independent chemostat populations studied by Helling et al. [4]. And in a majority of these (6 of 8 tested) acetate scavengers evolved and rose to frequencies easily detected by colony screening [22]. Both phenotypes therefore commonly arise under continuous glucose limitation, and can do so within a few hundred generations. It is noteworthy that even ancestral *E*. *coli* incompletely metabolizes glucose, and leaves appreciable levels of residual acetate 194±20 nmol mL^-1^ [40]. Thus, there is an immediate selective advantage to any mutant that can access this secondary resource. In the case of experiments where SCV glucose specialists arise, this selective advantage is amplified, as mutations conferring this phenotype can result in higher levels of residual acetate. Also, beneficial mutations that enable increased glucose assimilation may arise in a lineage before it acquires the capacity to scavenge acetate. This clearly happened in the Kinnersley et al. experiments [28]. Phylogenetic analyses suggest that the glucose specialist and acetate specialist clades diverged early in evolutionary process: they share only one derived Single Nucleotide Polymorphism, while they differ by hundreds of SNPs, including different mutations that increase expression of LamB glycoporin. One, a MalK mutant (103 D297E), gave rise to the glucose specialist (CV103), while another, a MalT (A53E) mutant, gave rise to descendants able to scavenge acetate and other overflow metabolites.

Recent work demonstrates that new beneficial alleles quickly proliferate under intense selection in nutrient-limited chemostats [41–43]. And in evolutionary experiments using the same ancestor described in [5], population re-sequencing data reveal that new selectively favored mutations can increase in frequency from 0 to > 0.90 in only 100 generations (Schwartz, Kinnersley, Sherlock and Rosenzweig *Personal communication*). While not an inevitable outcome of continuous glucose limited culture, acetate specialists may arise for a variety reasons, none mutually exclusive. For example, they may be favored at the onset, if sufficient residual acetate is present, or the causative mutations may hitchhike with novel alleles that enhance glucose transport. In the Helling et al. experiments the likelihood of these outcomes is increased by the facts that the ancestor is a mutator, and the key that unlocks the door to acetate scavenging by that ancestor is an unstable IS element in its *acs* operator [4].

But why don’t acetate specialists always emerge when overflow acetate creates a new niche [22]? Predictions of our model provide possible answers. In particular the model possesses multi-stable steady states (Fig 2a) which, by definition, means that for a given glucose concentration, small changes in the initial frequency of the acetate specialist can lead to dramatically different competition outcomes as illustrated in Fig 4. In general, density dependent multi-stable dynamics have been observed in a number of bacterial systems [29, 44, 45] where the authors reported dramatic differences in ecological and evolutionary outcomes between replicate populations.

**Fig 5:**
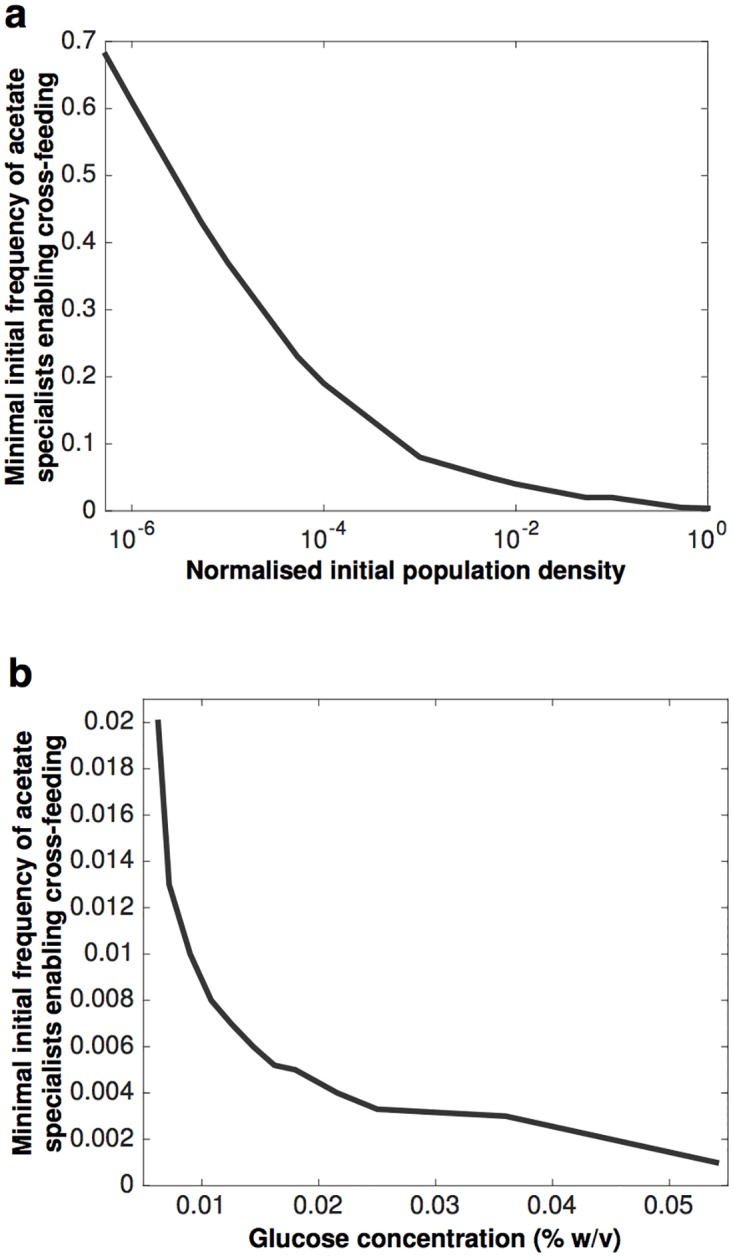
Model predictions. **(a)** The initial population size affects the success of acetate specialists. The higher the initial population size the lower the frequency of the acetate specialists at which this strain can invade and establish cross-feeding interactions. The initial population size is normalized with respect to the maximal population size supported on 0.025% glucose. **(b)** The glucose concentration in the input vessel (S_0_, % weight/volume) affects the success of acetate specialist. For a given glucose environment, the total initial population density is at the maximal level supported by that glucose concentration.

A direct consequence of multi-stability is the following observation: for a mutation that increases fitness via improved acetate assimilation to spread through a population, it has to emerge in a sufficiently large number of individuals. Given this, how can an acetate mutant ever become established? Interestingly, we find that the higher the initial population density in an environment, the lower the minimal and maximal initial frequencies of the acetate specialist for which cross-feeding interactions form (Figs 5a and 2b). After all, when the density of a glucose specialist, such as the genotype described in [28, 40], is increased by addition of glucose, there occurs a stoichiometric increase in concentration of the secondary resource, acetate. More amply supplied with this resource, a newly arisen acetate specialist can more rapidly increase to a higher frequency than it would otherwise, diminishing the likelihood that it fails to become established in the population. Consistent with this, our model also predicts that the higher the glucose concentration in the input vessel, the lower the initial frequency of acetate specialist has to be for the cross-feeding interactions to be observed (Fig 5b). This means that for example, for a sufficiently high glucose concentration in the input vessel our model requires a frequency of 0.002 of acetate mutants in a population for the cross-feeding to become established. We now know that JA122, the ancestral strain used as a founder by Helling et al. [4], was a mutator, owing to nonsense mutation in the mismatch repair enzyme, MutY (L299*) [28]. The mutation rate of JA122 is nearly two orders of magnitude higher than that of wild-type strain K12 (1.00 x 10^−7^ vs. 3.6 x 10^−9^ /cell/generation), making it feasible that mutation required for cross-feeding could arise with a frequency of 0.002 in a sufficiently large population of JA122.

Interestingly, relative to K12, JA122 acquired a regulatory mutation 93 nt upstream of *acs*, which encodes the acetate scavenging enzyme, acetyl CoA synthetase. In the system we have analyzed, abundant expression of *acs* has been traced to movement of a transposon, IS*30* (transposition rate ~5 x 10^−6^ /element/generation) [46]; in independent evolution experiments constitutive activation of *acs* occurred via IS*3* insertion mutations at -38 nt as well as via T➔ A transversions at -93 nt [22] Clearly, this system has the genetic potential to evolve cross-feeding; and indeed, where this potential has been realized secondary resource specialists arose early [28] and attained appreciable frequencies [22].

Our model also contains a critical point, predicting that small changes in environmental conditions can cause abrupt and irreversible shifts from cross-feeding interactions being established to not being possible (Fig 3). Critical points are a feature of many biological systems and recent studies showed that a combination of controlled laboratory experiments and mathematical models can be effective in determining the associated population dynamics [29, 47].

At first glance, the basis for cross-feeding interactions seems intuitively obvious. Even in simple homogeneous environments where population growth is limited by a single primary resource, multiple competitors can coexist if additional niches open up. However, our mathematical model shows that complex density and frequency dependence dynamics govern both the establishment and maintenance of these interactions. Given that such complex multi-stable dynamics of cross-feeding exist in a simple chemostat model like ours, we hypothesize that they would also be found in systems with additional complexities such as temporal heterogeneities [48]. Unlike the chemostat where resource concentration and cell densities are maintained at a constant level, temporal heterogeneities are a feature of seasonal environments in which the cell population and the primary resource from which it draws sustenance profoundly change each day. Indeed, a hallmark of bi-stable dynamics has been observed in seasonal environments [49] as only 1 out of 6 replicate clonal populations evolved strong cross-feeding interactions which were maintained in the long-term [50]. Moreover, a theoretical model based on [32] showed that the observed cross-feeding interactions in seasonal environments give rise to linear negative-frequency dependence [51].

The lack of cross-feeding polymorphism in seasonal compared to chemostat environments was predicted in theory [32], and it has been hypothesized that this could be driven by differences in cell densities and resource concentration between the two environments [48]. Namely, chemostat experimental studies where cross-feeding was observed [4, 5] supported both higher concentration of the primary resource and higher cell densities than seasonal experiments [49]. In addition, we hypothesize that density and frequency dependent bi-stable dynamics could also be an important feature of seasonal environments whereby seemingly similar environmental conditions can lead to very different outcomes with respect to the evolution of cross-feeding, consistent with observations in [49].

Our model can readily be extended to make testable predictions regarding the stability of cross-feeding interactions over evolutionary time. Prior simpler theoretical models suggest that if cross-feeding emerges in a clonal population, it will lead to extreme specialization with one strain specializing on the primary resource and the other on the secondary [32]. However, the cross-feeding that evolved between *E*. *coli* strains described in both [5] and [48] is facultative; partners can grow independently on the primary resource but clearly exist over hundreds of generations as co-evolving lineages [28, 52]. Experiments, informed by the modeling approach described here, can therefore be designed to address outstanding questions such as: how does this co-evolutionary process affect the boundary conditions for stable coexistence?, what are the pre-requisites for cross-feeding to evolve further into syntrophy (e.g. [53])?, and under what conditions might we expect such interactions to increase the metabolic efficiency of a co-evolving system (e.g. [54])?

The applications of our findings are broad. The origin and fate of genetic diversity are central organizing themes in biology, and trophic interactions may play a decisive role in promoting microbial diversity in extreme and/or nutrient-poor environments [55, 56]. Furthermore, cross-feeding has relevance for human health: trophic interactions of this nature could help subpopulations in tumors and chronic infections become genetically heterogenous, providing thereby more ways for cells to evolve drug resistance and to escape immune system surveillance. Lastly, because extracellular metabolites contain information and can be co-opted as signaling factors, cross-feeding can set the stage for communication between pathogenic species in polymicrobial infections [57]. In light of the stochastic fluctuations that inevitably occur in both laboratory and natural settings, we argue that mathematical models are essential tools for disentangling the complexities of cross-feeding.

## Supporting Information

S1 Supporting Information Model Parameterization and Experimental Methods for Parameter Estimation and Verification. Supporting Information file contains A. Model parameterization and B. Experimental methods for parameter estimation and verification.
(DOCX)
