# Supplementary material for: Stability of Cross-Feeding Polymorphisms in Microbial Communities
Source: PLoS Comput Biol. 2016 Dec 30;12(12):e1005269. doi: 10.1371/journal.pcbi.1005269 (PMC5201250; doi:10.1371/journal.pcbi.1005269)
Supplement: S1 Supporting Information — Supporting Information file contains A. Model parameterization and B. Experimental methods for parameter estimation and verification. (DOCX) [file pcbi.1005269.s001.docx]

**S1 Supporting Information. Model Parameterization and Experimental Methods for Parameter Estimation and Verification**

**A. Model parameterization**

We parameterize the model using two cross feeding strains of *E. coli* where *N_1_* denotes a glucose specialist (CV103) and *N_2_* denotes an acetate specialist (CV101) grown in glucose-limited chemostats (Rosenzweig *et al.* 1994).

CV103 takes up glucose at a rate that is 50% greater than CV101; however, glucose consumption is largely fermentative leading to excretion of acetate, the predominant by-product of *E. coli* fermentation (Rosenzweig *et al.* 1994; Kinnersley *et al.* 2014). During growth on glucose, a fraction of the glucose metabolized via pyruvate to acetyl-CoA is diverted and excreted out of the cell by a reversible reaction sequence involving acetate kinase (LeVine *et al.* 1980). The remaining glucose is fully metabolized through a TCA cycle. On the other hand while CV101 has inferior glucose uptake kinetics it secures preferential access to acetate excreted by CV103 as a result a promoter mutation in *acs* which encodes the acetate-scavenging enzyme acetyl CoA synthase (Kinnersley *et al.* 2014). The estimates for the yield of glycolysis (*n_g_*) and the TCA cycle (*n_tca_*) in our simple two-reaction metabolic representation of sugar catabolism are shown in Supplement Figure 2 and summarized in Supplement Table 1. For simplicity, as in (MacLean & Gudelj 2006), our model does not take into account the ATP production/usage for the acetate kinase step; this does not qualitatively alter the outcome. Importantly, even though our model coarse-grains the yield of sugar catabolism to two quantities *n_g_* and *n_tca_* the model outcomes are qualitatively robust to small changes in their values.

We assume that the rate of glucose uptake ($\boldsymbol{v}_{\boldsymbol{g,i}}$) for each of the two strains *i=1,2* show saturating enzyme kinetics of the form

$\boldsymbol{v}_{\boldsymbol{g,i}}\boldsymbol{(x)=}\frac{\boldsymbol{V}_{\boldsymbol{max,g,i}}\boldsymbol{\cdot x}}{\boldsymbol{K}_{\boldsymbol{m,g,i}}\boldsymbol{+x}}$

where $V_{max,g,i}$ denotes the maximum glucose uptake rate and $K_{m,g,i}$ denotes half saturation constant. The reversible transport rate of metabolic intermediates denoted by $\boldsymbol{v}_{\boldsymbol{rt,i}}$ in the model (1) is represented by the rate of acetate kinase and for each of the two strains *i=1,2* we assume it shows saturating enzyme kinetics of the form

$$\boldsymbol{v}_{\boldsymbol{rt,i}}\boldsymbol{(}\boldsymbol{X}_{\boldsymbol{ex}}\boldsymbol{-}\boldsymbol{X}_{\boldsymbol{in,i}}\boldsymbol{)=}\frac{\boldsymbol{V}_{\boldsymbol{max,ak,i}}\boldsymbol{\cdot(}\boldsymbol{X}_{\boldsymbol{ex}}\boldsymbol{-}\boldsymbol{X}_{\boldsymbol{in,i}}\boldsymbol{)}}{\boldsymbol{K}_{\boldsymbol{m,ak,i}}\boldsymbol{+}\left| \boldsymbol{X}_{\boldsymbol{ex}}\boldsymbol{-}\boldsymbol{X}_{\boldsymbol{in,i}} \right.\boldsymbol{|}}$$

where $V_{max,ak,i}$ represents the maximum rate of acetate kinase while $K_{m,ak,i}$ is the half saturation constant of acetate kinase. The variables$\boldsymbol{X}_{\boldsymbol{ex}}$ and $\boldsymbol{X}_{\boldsymbol{in,i}}$denote the concentration of the extracellular and intracellular metabolic intermediates, respectively. Note that since initially $\boldsymbol{X}_{\boldsymbol{ex}}=0$, the transport rate $\boldsymbol{v}_{\boldsymbol{rt,i}}$ is the main route out of the cell for the metabolic intermediates. The glycolysis and acetate kinase parameters have already been estimated in (Helling *et al.* 1987; Rosenzweig *et al.* 1994) for CV101 and CV103 and their values are listed in Supplement Table 1.

The acetate specialist strain (CV101) expresses high levels of the high affinity enzyme (ACS) for the uptake of acetate and the rate of this enzyme kinetics, denoted by $\boldsymbol{v}_{\boldsymbol{hat}}$ in the model (1), takes the form

$$\boldsymbol{v}_{\boldsymbol{hat}}\boldsymbol{(x)=}\frac{\boldsymbol{V}_{\boldsymbol{max,acs}}\boldsymbol{\cdot x}}{\boldsymbol{K}_{\boldsymbol{m,acs}}\boldsymbol{+x}}$$

where $V_{max,acs}$ denotes the maximum rate of ACS while $K_{m,acs}$ denotes the half-saturation constant of ACS. The maximum rate of ACS parameter has been previously reported in (Rosenzweig *et al.* 1994) and since no mutations were observed in the coding region of ACS (Kinnersley *et al.* 2014), it is reasonable to assume that the protein’s $K_{m,acs}$ values in a founder strain (JA122), acetate specialist (CV101) and glucose specialist (CV103) all approximate the previously reported wild-type values (Rosenzweig *et al.* 1994). Both $V_{max,acs}$ and $K_{m,acs}$ values are listed in Supplement Table 1. Also note that the change at ACS between CV101 and CV103 is a regulatory change arising from a promoter mutation found in CV101 but not in CV103.

Since citrate synthase is the rate-limiting enzyme of the TCA cycle when acetate is the limiting resource, we assume that the rate of TCA cycle ($\boldsymbol{v}_{\boldsymbol{tca}}\boldsymbol{)}$ is governed by the rate of citrate synthase. In particular we assume that

$$\boldsymbol{v}_{\boldsymbol{tca}}\left( \boldsymbol{x} \right)\boldsymbol{=}\frac{\boldsymbol{V}_{\boldsymbol{max,cs,i}}\boldsymbol{\cdot x}}{\boldsymbol{K}_{\boldsymbol{m,cs,i}}\boldsymbol{+x}}$$

where $\boldsymbol{V}_{\boldsymbol{max,cs,i}}$ denotes the maximum rate of citrate synthase while $\boldsymbol{K}_{\boldsymbol{m,cs,i}}$ is the half-saturation constant with $i=1,2$ referring to the two strains. We measured $\boldsymbol{V}_{\boldsymbol{max,cs,i}}$ for both CV103 and CV101 (Supplementary Information Part B. Experimental Methods: Experimental design A and Supplement Figure 3) and the values listed in Supplement Table 1. In addition we assume that $\boldsymbol{K}_{\boldsymbol{m,cs}}$ for both strains takes the same value as for the WT *E. coli* which was measured in (Molgat *et al.* 1992) and summarized in Supplement Table 1.

We assume that acetate imposes an inhibitory cost to growth ($\boldsymbol{c}_{\boldsymbol{i}}$) for both strains $i=1,2$ and this inhibition function takes the form (Davison & Stephanopoulos 1986)

$$\boldsymbol{c}_{\boldsymbol{i}}\boldsymbol{(x)=}\frac{\boldsymbol{1}}{\boldsymbol{1+}\frac{\boldsymbol{x}}{\boldsymbol{K}_{\boldsymbol{in,i}}}}$$

where $\boldsymbol{K}_{\boldsymbol{in,i}}$ is the inhibition constant, which is equivalent to the concentration of the inhibitor at which the specific growth rate is halved from its value with no inhibitor present.

We measured $\boldsymbol{K}_{\boldsymbol{in,i}}$ for both strains CV103 and CV101 (Supplementary Information Part B. Experimental Methods: Experimental design C) with the results shown in Supplement Figure 4 and the values summarized in the Supplement Table 1.

In order to connect cellular metabolism to growth we assume that rate of cell growth is proportional to the rate of ATP production (Bauchop & Elsden 1960) according to a proportionality constant G. We choose G (Supplement Table 1) so that the chemostat with D=0.003/min can support growth of each strain in isolation for a range of glucose concentrations (0.002% - 0.025%) as previously observed (Helling *et al.* 1987; Rosenzweig *et al.* 1994) and that the two strains can be maintained together at 0.00625% glucose concentrations as previously observed (Rosenzweig *et al.* 1994) but note that the model outcomes are robust to the changes in the value of G.

**B. Experimental Methods for Parameter Estimation and Verification**

**Conversion of soluble protein and g dry weight to cell numbers**

In order to ensure that all parameter values are expressed in the same units of measurement we determined conversion of soluble protein to g dry weight for each of the strains (Experimental design A). We found that on average: JA122 had 68.0 mg soluble protein / g dry weight, CV103 had 105.97 mg soluble protein / g dry weight while CV101 had 57.5 mg protein / g dry weight. We also assume that one *E. coli* cell weighs 3x10^-13^g (Bradford 1976).

**Experimental Design A: Determination of citrate synthase (CS) activity in batch culture and in glucose-limited chemostats**

*Cell culture*. Cells were aerobically cultured in glucose-limited (0.0125%) chemostats at 30^o^ in Davis minimal medium, as modified by (Helling *et al.* 1987), and harvested at steady state (A_550_= 0.098-0.137) after 72 h. Additionally, cells were aerobically batch cultured overnight at 30^o^ and grown to mid log phase (A_550_= 0.21-0.31) either in Davis minimal medium containing glucose (0.125 g/L) (CS repressing) and or in Davis medium containing acetate (0.17 g/L) (CS inducing). Experimental treatments were performed at least in duplicate. In each case, 50 mL of culture was harvested in duplicate by fast filtration over a 0.45 μM Millipore Nylon filter. Filters were transferred immediately to tubes containing liquid N_2_, placed at -80^o^C, then processed essentially as described (De Maeseneire *et al.* 2006) after no more than 48 h. Specifically, filtered cells were vortexed in 2 mL ice-cold Phosphate buffer (5 mM Phosphate buffer pH 7.5, 5 mM MgCl_2_, 1 mM EDTA) placed in an ice bath, then sonicated at 30% power in 4 x 15 s bursts with 15s vortexing intermittent (Branson Digital Sonifier 250, 1/8” microtip, 200W). Cell lysates were centrifuged 15 min at 5000 *x* *g* at 4^o^C in a Beckman Allegra centrifuge, then transferred to a clean -80^o^C chilled tube and stored at -80^o^. 200 μL of each lysate was reserved for assay of total soluble protein using Bradford reagent (B6916 Sigma) and bovine serum albumen as standard (Bradford 1976).

*Citrate Synthase determination* The specific activity of citrate synthase (oxaloaxcetate + acetyl CoA 🡪 citrate + CoA) was assayed spectrophotometrically at 25^o^ as described by (Morgunov & Srere 1998) by following the reaction of CoA with dithionitrobenzoate (DTNB), and measuring thiobenzoate, which absorbs maximally at 412nm (*ε=*13.6 x10^3^ M^-1^ cm^-1^). CS activity in cell lysates was determined in 96-well format using a Spectramax M2^e^ (Molecular Devices) plate reader, with sample volumes (2-20 μl) roughly adjusted for their protein concentration. Values were determined in triplicate using 0.2 mM acetyl-CoA, and 0.1mM DTNB in 50 mM HEPES, 2 mM EDTA, 100 mM KCl. Background activity was measured spectrophotometrically at 412 nm for 5 min, then CS activity measured for 7 min following the addition of 10 μl of 10 mM OAA (oxaloacetate). Estimates of specific activity were made over a linear interval of > 7 min; reaction rate was calculated over 5 min as per (Srere 1969) and reported as U/mg protein.

**Experimental design B: Measurement of NADH/NAD+ (redox) ratio in aerobic and in anaerobic glucose-limited chemostat cultures**

For NADH/NAD determination, *E. coli* were grown to steady-state in glucose-limited chemostats as previously described (Kinnersley *et al.* 2014). For anaerobic determinations, aerobically propagated steady-state cultures were shifted to anaerobiosis by continuously bubbling with N_2_ for 1.5 hours prior to harvest. Cells were harvested directly into a protective buffer of 0.2N NaOH with 1% DTAB (Dodecyltrimethylammonium bromide) and processed immediately. Nucleotides were extracted through three cycles of freeze-thaw lysis, then quantified using the NAD/NADH-Glo assay kit per manufacturer’s instructions (Promega, Madison, WI**,** <https://www.promega.com/-/media/files/resources/protocols/technical-manuals/101/nad-nadh-glo-assay-protocol.pdf>). Relative luminescence ratios calculated from triplicate measures on each of three independent chemostats for each strain/condition were averaged and are presented with error bars corresponding to ± 1 SEM.

**Experimental design C: Determination of Ki (inhibition constant) for acetate**

Acetate inhibition studies were conducted in 96-well plates in Davis minimal medium supplemented with 0.0125% glucose and either 0, 0.5, 1, 1.5 or 2 g/L acetate (Helling *et al.* 1981). JA122, CV101, CV103 and CV116 were grown from glycerol stock on TA (tryptone agar) plates, collected with a sterile cotton swab to avoid carryover of media, and re-suspended in Davis minimal salts lacking a carbon source. Each strain was assayed in triplicate for each acetate concentration in 100 μl volumes on a single 96-well plate. Absorbance readings (550 nm) were collected every 10 minutes over 30 hours at 30°C with constant shaking using a Biotek Synergy MX plate reader (Winooski, VT). Maximum specific growth rates (μ_max_) were calculated from absorbance data using Curve Fitter (Nigel Delaney, <http://www.evolvedmicrobe.com/CurveFitter/>). Average *μ_max_* for triplicate cultures was plotted vs. acetate concentration, and the inhibition constants (K^I^) were calculated from the best-fit line as the acetate concentration at which the measured growth rate was ½ that of cells grown in the absence of acetate (Davison & Stephanopoulos 1986).

**References**

1. Bauchop, T. & Elsden, S.R. (1960). The growth of micro-organisms in relation to their energy supply. *Journal of general microbiology*, 23, 457-469. (Reference [26] in main text).

2. Bradford, M.M. (1976). A rapid and sensitive method for the quantitation of microgram quantities of protein utilizing the principle of protein-dye binding. *Analytical biochemistry*, 72, 248-254.

3. Davison, B.H. & Stephanopoulos, G. (1986). Coexistence of S. cerevisiae and E. coli in a Chemostat under Substrate Competition and Product Inhibition. *Biotecnology and Bioengineering*, XXVIII, 1742-1752. (Reference [27] in main text).

4. De Maeseneire, S.L., De Mey, M., Vandedrinck, S. & Vandamme, E.J. (2006). Metabolic characterisation of E. coli citrate synthase and phosphoenolpyruvate carboxylase mutants in aerobic cultures. *Biotechnology letters*, 28, 1945-1953.

5. Helling, R.B., Kinney, T. & Adams, J. (1981). The maintenance of Plasmid-containing organisms in populations of Escherichia coli. *J Gen Microbiol*, 123, 129-141.

6. Helling, R.B., Vargas, C.N. & Adams, J. (1987). Evolution of Escherichia coli During Growth in a Constant Environment. *Genetics*, 116, 349-358. (Reference [4] in main text).

7. Kinnersley, M., Wenger, J., Kroll, E., Adams, J., Sherlock, G. & Rosenzweig, F. (2014). Ex uno plures: clonal reinforcement drives evolution of a simple microbial community. *PLoS Genetics*, 10. (Reference [28] in main text).

8. LeVine, S.M., Ardeshir, F. & Ames, G.F. (1980). Isolation and Characterization of acetate kinase and phosphotransacetylase mutants of Escherichia coli and Salmonella typhimurium. *J Bacteriol*, 143, 1081-1085.

9. MacLean, R.C. & Gudelj, I. (2006). Resource competition and social conflict in experimental populations of yeast. *Nature*, 441, 498-501. (Reference [25] in main text).

10. Molgat, G.F., Donald Lj Fau - Duckworth, H.W. & Duckworth, H.W. (1992). Chimeric allosteric citrate synthases: construction and properties of citrate synthases containing domains from two different enzymes. *Archives of Biochemistry and Biophysics*, 298(1).

11. Morgunov, I. & Srere, P.A. (1998). Interaction between citrate synthase and malate dehydrogenase. Substrate channeling of oxaloacetate. *The Journal of biological chemistry*, 273, 29540-29544.

12. Rosenzweig, R.F., Sharp, R.R., Treves, D.S. & Adams, J. (1994). Microbial Evolution. *Genetics*, 137, 903-917. (Reference [5] in main text).

13. Srere, P.A. (1969). [1] Citrate synthase: [EC 4.1.3.7. Citrate oxaloacetate-lyase (CoA-acetylating)]. In: *Methods in Enzymology* (ed. John, ML). Academic Press, pp. 3-11.

**Supplement Figure 1a**

Model predictions of the initial acetate secretion by the acetate specialist (CV101) and glucose specialist (CV103) strains grown in isolation on 0.00625% glucose in the chemostat.

**Supplement Figure 1b**

To determine the redox ratio (NADH/NAD), strains were grown to steady state in glucose-limited chemostats. For anaerobic determinations aerobically propagated steady-state cultures were shifted to anaerobiosis for 1.5 hours prior to harvest. Cells were harvested and nucleotides extracted immediately, with relative luminescence ration calculated from triplicate measures on each of three independent chemostats for each strain/condition. Average values are shown in the figure below (mean 1±SEM).

**Supplement Figure 1c**

Model predictions of acetate secretion by the glucose specialist, CV103 strain, grown in the chemostat environments with different glucose concentrations.

**Supplement Figure 2:** Estimates of the yield of glycolysis (*n_g_*) and the TCA cycle (*n_tca_*) in our simple two-reaction metabolic representation of sugar catabolism (equation (1) of the main text).

**Supplement Figure 3:** The specific activity of citrate synthase for each strain JA122, CV101 and CV103 in the glucose limited chemostat, was assayed spectrophotometrically at 25° by following reaction of CoA with dithionitrobenzoate (DTNB) and measuring thiobenzoate. CS activity in cell lysates was determined in 96-well format using a plate reader (mean 1±SEM, N=2).

**Supplement Figure 4:** Acetate inhibition constant from the three strains CV101, CV103 and JA122 was estimated in the following way. Strains were assayed in triplicates for different acetate concentrations (0-2g/L) on a single 96-well plate measuring absorbance over 30 hours. This data was used to estimate the maximal growth rate as a function of acetate concentrations for each of the strains, which is shown in the figure below. Linear curves were fitted to maximum growth rate vs. acetate concentration data for each strain (R^2^=0.97 for CV101; R^2^=0.67 for CV103 and R^2^=0.96 for JA122) and these linear approximations used to determine an acetate concentration at which the maximal growth rate halved from its value with no acetate present in the environment. This measure is called acetate inhibition constant KI.

**Supplement Table 1**

| Parameter description | Parameter notation | Parameter value | Reference |
| --- | --- | --- | --- |
| Maximal glucose uptake rate for *N_1_* | $\boldsymbol{V}_{\boldsymbol{max,g,1}}$ | 7.38x10^-13^ μmol/min/cell | (Rosenzweig *et al.* 1994) |
| Maximal glucose uptake rate for *N_2_* | $\boldsymbol{V}_{\boldsymbol{max,g,2}}$ | 4.98x10^-13^ μmol/min/cell | (Rosenzweig *et al.* 1994) |
| Half saturation constant of glucose uptake for *N_1_* | $\boldsymbol{K}_{\boldsymbol{m,g,1}}$ | 0.01x10^3^ μmol/L | (Rosenzweig *et al.* 1994) |
| Half saturation constant of glucose uptake for *N_2_* | $\boldsymbol{K}_{\boldsymbol{m,g,2}}$ | 0.01x10^3^ μmol/L | **(R**osenzweig *et al.* 1994) |
| Maximum rate of acetate kinase for *N_1_* | $\boldsymbol{V}_{\boldsymbol{max,ak,1}}$ | 376.7x10^-13^ μmol/min/cell | (Rosenzweig *et al.* 1994) |
| Maximum rate of acetate kinase for *N_2_* | $\boldsymbol{V}_{\boldsymbol{max,ak,2}}$ | 205x10^-13^ μmol/min/cell | (Rosenzweig *et al.* 1994) |
| Half saturation constant of acetate kinase for *N_1_* | $\boldsymbol{K}_{\boldsymbol{m,ak,1}}$ | 14x10^3^ μmol/L | (Rosenzweig *et al.* 1994) |
| Half saturation constant of acetate kinase for *N_2_* | $\boldsymbol{K}_{\boldsymbol{m,ak,2}}$ | 32x10^3^ μmol/L | (Rosenzweig *et al.* 1994) |
| Maximum rate of ACS for *N_2_* | $\boldsymbol{V}_{\boldsymbol{max,acs}}$ | 21x10^-13^ μmol/min/cell | (Rosenzweig *et al.* 1994) |
| Half saturation constant of ACS for *N_2_* | $\boldsymbol{K}_{\boldsymbol{m,acs}}$ | 200 μmol/L | (Rosenzweig *et al.* 1994) |
| Maximum rate of citrate synthase for *N_1_* | $\boldsymbol{V}_{\boldsymbol{max,cs,1}}$ | 27x10^-13^ μmol/min/cell | This study |
| Maximum rate of citrate synthase for *N_2_* | $\boldsymbol{V}_{\boldsymbol{max,cs,2}}$ | 28x10^-13^ μmol/min/cell | This study |
| Half saturation constant of citrate synthase for *N_1_* | $\boldsymbol{K}_{\boldsymbol{m,cs,1}}$ | 700 μmol/L | [Molgat *et al.* 1992](#_ENREF_16)**)** |
| Half saturation constant of citrate synthase for *N_2_* | $\boldsymbol{K}_{\boldsymbol{m,cs,2}}$ | 700 μmol/L | [Molgat *et al.* 1992](#_ENREF_16)**)** |
| Acetate inhibition constant for *N_1_* | $\boldsymbol{K}_{\boldsymbol{in,1}}$ | 10^5^ μmol/L | This study |
| Acetate inhibition constant for *N_2_* | $\boldsymbol{K}_{\boldsymbol{in,2}}$ | 4x10^4^ μmol/L | This study |
| Chemastat dilution rate | $\boldsymbol{D}$ | 0.003/min | (Rosenzweig *et al.* 1994) |
| Yield of glycolysis | $\boldsymbol{n}_{\boldsymbol{g}}$ | 12 μmol ATP/ μmol of *S* | This study |
| Yield of TCA | $\boldsymbol{n}_{\boldsymbol{tca}}$ | 12 μmol ATP/ μmol of *X_in_* | This study |
| Growth proportionality constant | $\boldsymbol{G}$ | 3.75x10^9^ cells/ μmol ATP | This study |
